# Modeling strategic use of human computer interfaces with novel hidden Markov models

**DOI:** 10.3389/fpsyg.2015.00919

**Published:** 2015-07-03

**Authors:** Laura J. Mariano, Joshua C. Poore, David M. Krum, Jana L. Schwartz, William D. Coskren, Eric M. Jones

**Affiliations:** ^1^The Charles Stark Draper Laboratory, Inc.Cambridge, MA, USA; ^2^Mixed Reality Lab, Institute for Creative Technologies, University of Southern CaliforniaLos Angeles, CA, USA

**Keywords:** virtual environments, behavioral modeling, UX, Markov processes, contextual computing, human computer interaction, hidden Markov models

## Abstract

Immersive software tools are virtual environments designed to give their users an augmented view of real-world data and ways of manipulating that data. As virtual environments, every action users make while interacting with these tools can be carefully logged, as can the state of the software and the information it presents to the user, giving these actions context. This data provides a high-resolution lens through which dynamic cognitive and behavioral processes can be viewed. In this report, we describe new methods for the analysis and interpretation of such data, utilizing a novel implementation of the Beta Process Hidden Markov Model (BP-HMM) for analysis of software activity logs. We further report the results of a preliminary study designed to establish the validity of our modeling approach. A group of 20 participants were asked to play a simple computer game, instrumented to log every interaction with the interface. Participants had no previous experience with the game's functionality or rules, so the activity logs collected during their naïve interactions capture patterns of exploratory behavior and skill acquisition as they attempted to learn the rules of the game. Pre- and post-task questionnaires probed for self-reported styles of problem solving, as well as task engagement, difficulty, and workload. We jointly modeled the activity log sequences collected from all participants using the BP-HMM approach, identifying a global library of activity patterns representative of the collective behavior of all the participants. Analyses show systematic relationships between both pre- and post-task questionnaires, self-reported approaches to analytic problem solving, and metrics extracted from the BP-HMM decomposition. Overall, we find that this novel approach to decomposing unstructured behavioral data within software environments provides a sensible means for understanding how users learn to integrate software functionality for strategic task pursuit.

## Introduction

Software logs collected during users' interactions with a virtual environment provide a complex, dynamic data source from which patterns of human behavior can be mined. By treating software as the sensor, users' behavior is constrained only by the limitations of the virtual environment, enabling collection of natural behavior patterns in real-time. Such data provides a high definition lens through which dynamic cognitive and behavioral processes can be viewed, making the software-as-sensor experimental paradigm a powerful tool for the study of such behaviors. In this report, we describe new methods for the analysis and interpretation of such data, utilizing a novel implementation of the Beta-Process Hidden Markov Model (BP-HMM) to model software activity logs collected from individuals playing a simple computer game which they have never seen before. We also offer experimental validation that this modeling approach was able to capture meaningful variation in the actual behaviors encoded by these logs.

HMMs have long been used to characterize the dynamics of time-series data in a wide variety of domains, including speech recognition (Gales and Young, 2008), gesture recognition (Mitra and Acharya, 2007), and bioinformatics (Yoon, 2009). An HMM is a type of Markov model that represents the dynamics of a stochastic system as a set of states and state transition probabilities, where the states themselves are not observable. A data (observation) sequence is generated by an HMM according to the set of observation probability distributions associated with the hidden states, and the state transition probabilities of the model, both of which are learned from the data (Rabiner and Juang, 1986). Our selection of an HMM-based approach for modeling software activity logs was motivated by the hypothesis that canonical behavior patterns in the logs could be quantified by the parameters of an HMM representation of the data sequences. The properties of the hidden state observation probability distributions represent distinctly different “modes” of behavior as distributions over the frequencies of temporal co-occurrence of the activity logs. The state transition probabilities capture the dynamics of how these behavior modes interact.

The use of HMMs for the analysis of software activity logs is not without precedent, and has proven successful in other domains, including analysis of click-stream data for assessment of web-browsing behavior (Ypma and Heskes, 2003; Laxman et al., 2008; Schwartz et al., 2011; Melnykov, 2014), and for identification of insider-threat patterns in computer usage and database access logs (Thompson, 2004; Ted et al., 2013). Traditional HMMs are designed to represent the dynamics of a single underlying Markov process. Methods for extending single-sequence HMMs to more complex, multi-series datasets include coupled HMMs (Brand et al., 1997), and mixed HMMs (Altman, 2007), which are utilized in several click-stream analysis studies (Ypma and Heskes, 2003; Melnykov, 2014). Similarly, in this study we were interested in identifying canonical behavior patterns in the ensemble of software logs, utilizing the BP-HMM approach to multi-sequence modeling. The BP-HMM identifies a global library of activity patterns (states) shared by an ensemble of related data sequences, and assigns a subset of these behaviors to each sequence individually. Each sequence is modeled as an HMM, but the states populating these models are pulled from a global pool, allowing for direct comparisons between the models (Fox et al., 2009). This means that data from different individuals or from the same individual across multiple sessions can be described using the same state-space. Using the BP-HMM we were able to identify canonical behavior patterns in the software logs representative of learning, skill-acquisition, and mastery of the game. To our knowledge, this study is the first to apply the BP-HMM method to software activity logs. It has previously been used for the discovery of activity patterns from video collections (Hughes and Sudderth, 2012).

In this methodology report, we illustrate how the BP-HMM can be applied to the analysis of sequential behavior within software environments. Sequential behavior was captured as part of a pilot experiment during which participants interacted with a simple computer game for the first time. Participants had to learn to play the game and acquire points through self-driven, unstructured exploration of the game's interface. Using data collected from this experiment, we demonstrate that quantitative metrics extracted from modeling output contain information related to performance and user experiences. Finally, we discuss practical applications of the BP-HMM method for analysis of data in unstructured virtual environments, software-based task analysis, usability evaluation, and dynamic software adaptation.

## Materials and methods

### Participants

Participants were 20 members of the Cambridge, MA community, recruited through online advertisements (Craigslist). Participants were eligible on the basis that they were able to normally perceive color and whether they had either normal or corrected vision. Prior to engaging in laboratory tasks, all participants were distributed a consent form and intake questionnaire online. Participants who acknowledged the online consent form, and completed intake questionnaires were invited to the laboratory for testing procedures. Once in the laboratory, participants gave signed informed consent. All methods were approved by the New England Institutional Review Board (NEIRB; Protocol # 13–401). Of the 20 participants, 42% were female, 47% identified as Caucasian while the other half of participants identified as Asian (5%), Hispanic (5%), of African descent (~26%), and Indian (~11%); the remaining participants declined to state. On average, participants were 33.53 years of age (Range = 37 years, *SD* = 11.25 years). The sample was generally educated above the high-school level with most reporting a B.A. level education (68%), and only 5% reporting a M.A. level education.

### Laboratory procedures

#### Intake questionnaires

Once enrolled in the study each participant was distributed an intake questionnaire, hosted by surveymonkey.com. Questionnaires contained demographic items (age, ethnicity, education, etc.), as well as measures of analytic aptitude. The latter included; self-reports for the ability to use math in everyday situations [Subjective Numeracy Scale (SNS); Fagerlin et al., 2007]; and analytic problems that require algebraic formulation to solve, where an intuitive answer is almost certainly incorrect (Cognitive Reflections Test; Frederick, 2005); publically available, analytic questions involving sequencing or algebraic problem solving, in the style of Scholastic Aptitude Test (SAT) problems.

Questionnaires also solicited responses to measures of problem solving approach and cognitive style, including the rational-experiential inventory (Epstein et al., 1996), which gauges a propensity for deductive (rational) or inductive (experiential) problem solving styles. It also included the Need for Cognition scale (Cacioppo et al., 1984), which measures participants' desire for challenging cognitive problems, and the Need for Cognitive Closure scale (Webster and Kruglanski, 1994; Roets and Van Hiel, 2011), which addresses whether participants are willing to “settle” for answers to challenging problems for the sake of closure. Finally, the intake assessment included the Maximization Scale (Nenkov et al., 2008), which accounts for individuals' likelihood to strive for the best answer or alternative to a problem, at the expense of time. These items have been previously informative in studies regarding analytic problem solving (Poore et al., 2012, 2014). All measures were selected given their relevance to information foraging and analytic decision-making. Scaling, completion rates, and descriptive statistics for intake questionnaire measures are given in Supplementary Material (Tables 1–7), as well as cross correlations between questionnaire measures, task dependent variables and modeling output.

#### Data collection

**Figure 1:**
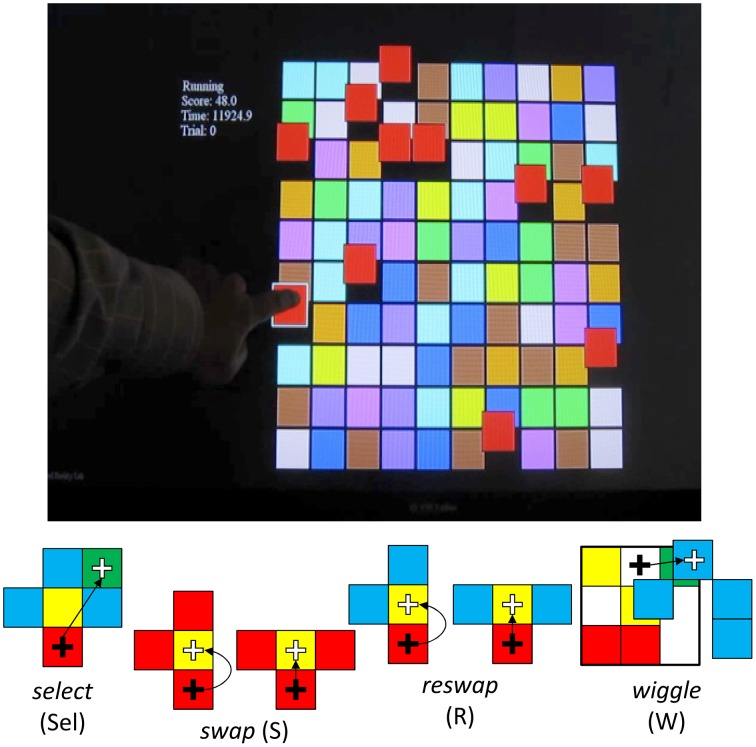
**USC-ICT's “Wiggle” Demonstration Game. Top panel**. The game presents users with a 10 × 10 matrix of colored tiles. Users are required to form groups of 3 adjacent sets of the same colored tiles. Doing so provides points and produces a chime. **Bottom Panel**: Users are constrained in how they can interact with the tiles. They can *select* a tile by tapping on it, and attempt to move that tile to an adjacent location by tapping on that location. If this move results in a grouping of 3 tiles of the same color, the user has made a *swap*, which scores points, and causes the entire matrix configuration to change. If the attempted move is to an adjacent location, and does not complete the pattern of three, this is treated like a failed swap attempt, and categorized as a *reswap*. This activity doesn't change the interface. Finally, users can also *wiggle* the tiles on the board by selecting a tile and dragging it to the side. This move causes all tiles of the same color to move with it, revealing spatial relationships between all the tiles of the same color on the board. This function is designed to aid in searching for swap opportunities. Participants are given 10 min to complete this task.

Having completed intake questionnaires, participants were invited to the Draper Laboratory (Cambridge, MA) for a 1 h session to complete tasks involving human computer interactions. Each participant was asked to play two sessions of a game called Wiggle, developed by the University of Southern California, Institute for Creative Technologies (USC-ICT; Ware and Bobrow, 2004, 2005) (see Figure 1; top panel). The game provides users with a 10 × 10 matrix of colored tiles. The goal of this game is to maneuver tiles into groups of 3 of the same color, using a finite set of acceptable moves (see Figure 1; bottom panel). Creating groups of 3 like-colored tiles scores points for the player. This game was presented to participants on a Hewlett-Packard touchscreen desktop computer—all participants interacted with the game exclusively with touchscreen inputs.

In order to manipulate the tiles presented on the interface, users can either *select* (Sel) a tile by tapping it, or they can drag it to the side, which is called a *wiggle* (W). When the user *wiggles*, all tiles in the grid of the same color move with it, revealing the spatial relationships between all tiles of that color. In order to move a tile, the player must first select the one they want to move, and then tap the location they want to move it to. If the new location creates a group of three tiles of the same color, this event is labeled a *swap* (S). If not, none of the blocks actually move, and the event is logged as a *reswap* (R).

The goal in using this game for the present study was to examine the different strategies participants might take in interacting with the game. As such, no participants were told exactly how to play the game or how to interact with the colored tiles. Instead, half of participants were not told anything about how to play the game, save that their task was to learn how to play the game. The other half were given some instruction that the goal of the game was to “score points by grouping like objects,” but they were not told how to do so. Additionally, half of participants were given a version of the game that enabled the wiggle function, but were not told about this function or how to use it. The other half of the participants interacted with the game without this feature. Participants played the game in two sessions, each lasting 10 min. This resulted in a mixed between and within subjects 2 [goal (instruction) vs. no goal (no instruction)] × 2 (wiggle vs. static) x 2 (session) research design; participants were randomly assigned to conditions.

#### Post-session questionnaires

Following each session, participants filled out a brief questionnaire asking them about their experiences with the game. Questionnaires included an abbreviated version of the Bedford workload measure (Roscoe and Ellis, 1990), including questions pertaining to self-reported task difficulty and mental effort, as well as game engagement (Brockmyer et al., 2009; Procci and Bowers, 2011). As part of this questionnaire, participants were given an opportunity to report what they thought the rules of the game were, using a free-response format.

### Behavioral data collection and extraction

The Wiggle software incorporates an activity logger which generates a time-stamped sequence of activity codes for each game-play session, where each activity code (i.e., Sel, S, R, W) corresponds to one of the four activities described above (i.e., *select*, *swap*, *reswap*, *wiggle*). This software instrumentation allows us to generate a time-series of participants' activities for each of their sessions, of which there were 40 total across participants. The raw time-series of activity sequences were then passed through a parser designed to extract context from some of the sub-sequences in the dataset. For example, a potential attempt to move a block must be preceded by selection of the block to be moved, so we reinterpreted rapid sequences of *select*-*wiggle*, *select*-*swap*, and *select*-*reswap* completed within 50 ms as simply *wiggle*, *swap*, and *reswap*, respectively. Since we were interested in understanding the sequence of actions the player intended to make, we felt that including the context surrounding activities would confound the intent of behaviors (e.g., *swap*) with the coded sequence used to implement that intent (e.g., *select*-*swap*).

**Figure 2:**
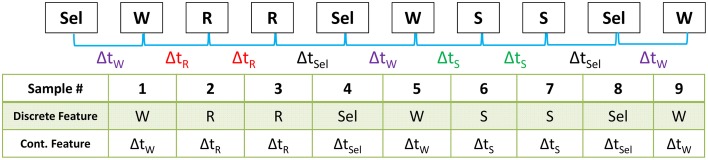
**The Wiggle computer game logged the sequence of user activities using 4 behavior codes (*select*, *swap*, *reswap*, and *wiggle*)**. We derived a second feature from this time series that is representative of the rate at which these behaviors occurred by computing the time delta between activities. At the top of the diagram is an example of a raw data sequence. The row of the table labeled “Categorical Feature” contains the sequence of behavior codes derived from the raw data, and the bottom row of the table contains the corresponding time delta between the previous and current activity. This process created the mixed categorical-continuous time-series dataset used in this analysis.

Using the re-coded sequences, we extracted an additional feature from the data. For each logged activity, we computed the time delay between the current and previous activity, generating a continuous-valued feature vector capturing the rate at which each activity was performed. Figure 2 depicts this process. Combining the temporal feature with the activity logs generated a 2-dimensional mixed-type data sequence with categorical and continuous components.

### Modeling user behavior

The entire ensemble of data sequences was jointly modeled as a single BP-HMM. In the following, we present a general overview of HMMs, the interpretation of its model parameters, and extension to ensemble modeling with BP-HMMs.

#### Hidden markov models

A Markov model represents the dynamics of a stochastic system as a set of states and state transition probabilities. At any given time, the system can be described as being in one of Kstates, *s*_1_, *s*_2_, …, *s*_3_ and the probability of transitioning between states is conditioned on the current state of the system. An HMM also assumes Markovian state transition dynamics, but the states themselves are not observable. Instead, the observations (data sequence) are a probabilistic function of *hidden* states, which emit the observations according to their individual observation probability distributions. A *K*-state first order discrete HMM is fully characterized by the following set of parameters:

- The set of M possible observations (symbols) generated by the underlying discrete process, described as:
  $$V=\left\{v_{1},v_{2},\;\ldots ,v_{M}\right\}$$
- The set of *K* hidden states in the model:
  $$S=\left\{s_{k}\right\},\;1\leq k\leq K$$
- The state transition probability matrix, *A*, containing the probabilities of transitioning from every state in the model to every other state. If the current state of the system at time *t* is *q_t_*:
  $$a_{ij}=P\left(q_{t+1}=s_{j}|q_{t}=s_{i}\right),0\leq a_{ij}\leq 1,\sum_{j=1}^{N}a_{ij}\;=1$$

The matrix *A* = {*a_ij_*} *for* 1 ≤ *i*, *j* ≤ *K*.

- The set of observation probability distributions, θ, for each of the hidden states. The discrete HMM is designed to capture the dynamics of a sequence of symbols drawn from a finite library of *M* possible observations. The form of the observation probability distributions of each hidden state is therefore an *M*-dimensional categorical distribution. For a given state *k*:
  $$\theta_{k}\left(i\right)=P\left(v_{i}\text{ at time }t|q_{t}=s_{k}\right),1\leq i\leq M$$

The parameters of each distribution, θ*_k_*(*i*) for 1 ≤ *k* ≤ *K* consist of the set of {*p*_1_, *p*_2_, …, *p_M_*}_*k*_ event probabilities, where $\sum_{i=1}^{M}p_{i}=1$. *p_i_* can be interpreted as the likelihood of observing symbol i while the system is in state k.

- The set of initial state distributions, *Z* = {*z_k_*}, where
  $$z_{k}=P\left(q_{1}=s_{k}\right),1\leq k\leq K$$

**Figure 3:**
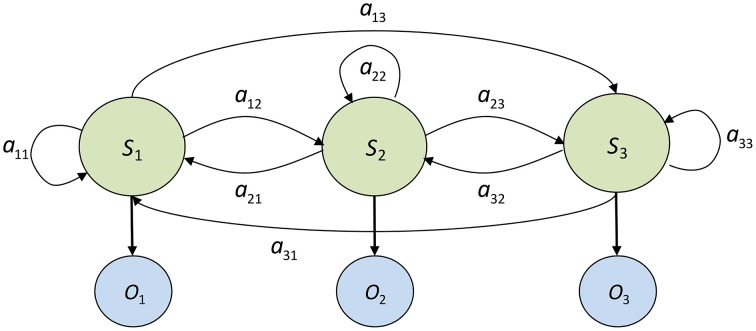
**Diagram of a fully connected (ergodic) 3-state Hidden Markov Model**. *S*_1_, *S*_2_, and *S*_3_ represent the hidden states, *O*_1_, *O*_2_, and *O*_3_ are observations emitted by the hidden states, and *a_ij_* represents the probability of transitioning from state *i* to state *j*.

Figure 3 graphically depicts the structure of a 3-state, fully connected HMM. *S*_1_, *S*_2_, and *S*_3_ represent the three hidden states, and *O*_1_, *O*_2_, and *O*_3_ are random variables representing the observations emitted by each state according to its emission probability distribution.

A key assumption we made when choosing to model the data sequences with HMMs is that a first-order Markovian representation of the underlying dynamics of the stochastic process is adequate—the probability of transition to a future state is conditioned only on the current state, and none that preceded it. We felt that this was a fair assumption to make given the nature of the data, but concede that there could be higher-order underlying dynamics in the data.

#### The beta-process hidden markov model

The BP-HMM can be thought of as a kind of latent feature model, where the “features” represent a (potentially unbounded) set of system states, and each data sequence has been generated by an HMM populated by some subset of these states, with sequence-specific state transition dynamics. Each sequence is modeled as an HMM, but the states populating these models are pulled from a global pool, allowing for direct comparisons between the models (Fox et al., 2009). The full set of parameters defining the BP-HMM with a library of K shared patterns (features), learned from an ensemble of *N* sequences is:

- The set of sparse binary vectors $f^{\left(i\right)}=\left[f_{1}^{\left(i\right)},f_{2}^{\left(i\right)},\ldots ,f_{K}^{\left(i\right)}\right]$, for 1 ≤ *i* ≤ *N*, indicating the presence or absence of each of the K features in the *i^th^* sequence. A value of ‘1’ in this vector indicates that the feature is ‘active’ in the sequence's HMM. The rows of the binary N x K matrix F contain the indicator vectors for the entire ensemble.
- The set of Kobservation probability distributions θ = {θ_1_, θ_2_, …, θ_*K*_}. Each θ_k_ is a categorical distribution composed of the set of {*p*_*k*1_, *p*_*k*2_, …, *p_kM_*} event probabilities, where $\sum_{i=1}^{M}p_{ki}=1$.
- The set of N state transition matrices, $\Pi =\left\{\pi^{\left(1\right)},\pi^{\left(2\right)},\ldots ,\pi^{\left(N\right)}\right\}$. Since the state transition matrices are sequence-specific, the size and interpretation of the elements of each matrix differ according to which features are active in that sequence. For a sequence π^(*i*)^ with L active features, π^(*i*)^ will be an *L x L* matrix whose rows and columns contain the probabilities of transitioning between these states. For this analysis, we chose to use sequence-specific state transition matrices.

The set of features assigned to each sequence is encoded in the *N x K* sparse binary indicator matrix, *F*, which is a member of the infinite set of binary matrices with *N* rows and a potentially infinite number of columns. The beta process is a stochastic process that can be used to identify the cardinality and structure of *F*, based on the underlying dynamics of the latent feature model.

Let *b_k_* be the ensemble-wide frequency of occurrence of feature *k*, and θ_*k*_ represent the data generating model corresponding to this feature. The global variables controlling the distribution of the latent features across the ensemble, and the properties of each feature, are generated by an underlying stochastic process, the beta process (BP):

$$B|B_{0},\gamma ,\beta \sim BP\left(\beta ,\gamma B_{0}\right),B=\sum_{k=1}^{\infty }b_{k}\delta_{\theta_{k}}$$

The random variable, *B* is drawn from the BP; it is the set of weights defining the inclusion probability of each feature in the model. The BP is parameterized by a mass parameter, γ, which influences the total number of active features in the ensemble, and the concentration parameter, β, which influences how those features are distributed across sequences in the ensemble. For each feature, θ_*k*_ ~ *B*_0_ marks its data generation parameters (Hughes et al., 2012). Each column of the binary matrix *F* is generated by a sequence of independent Bernoulli draws, *f_ik_* ~ *Ber* (*b_k_*), for *i* = 1, 2, …, *N*.

Fox et al. first described the application of the beta process prior to an ensemble of time-series data, where the features were autoregressive processes, and each sequence was modeled as an AR-HMM (the BP-AR-HMM) (Fox et al., 2009). In this work, we implemented the version of the BP-HMM described in Hughes and Sudderth (2012), where the data-generating parameters of each feature (θ_*k*_) are multinomial distributions of V activity codes, and each sequence is modeled as an HMM populated by a subset of these features, with sequence specific state transition dynamics. We used the *V*-dimensional symmetric Dirichlet distribution, with concentration parameter λ_0_ as the conjugate prior of the multinomial distributions:

$$\theta_{k}|B_{0}\sim Dir\left(\lambda_{0},\lambda_{0},\ldots ,\lambda_{0}\right)$$

We converted the final multinomial distributions describing each state into categorical distributions by normalizing over the total number of observations, which allowed us to more easily interpret the parameters as activity rates instead of raw activity counts.

As in Fox et al. (2009), the transition distributions are feature-constrained: $\pi^{\left(i\right)}=\left\{\pi_{k}^{\left(i\right)}\right\}$. Each π^(*i*)^ contains the parameters governing the *i^th^* object's transition probabilities among its active features. To obtain π^(*i*)^_*j*_ from each state *j* for sequence i, a set of individual transition weights, η^(*i*)^, is drawn, and then normalized by the number of active features assigned to sequence *i*:

$$\eta_{jk}^{\left(i\right)}|\alpha ,\kappa \sim Gamma\left(\alpha +\kappa \delta \left(j,k\right),1\right),\pi_{j}^{\left(i\right)}=\frac{\left[\begin{array}{ccc} \eta_{j1}^{\left(i\right)} & \eta_{j2}^{\left(i\right)} & \ldots \end{array}\right]⊗f_{i}}{\sum_{k|f_{ik=1}}\eta_{k1}^{\left(i\right)}},$$

where δ (*j*, *k*) is the Kronecker delta function, equal to 1 if *j* = *k*. *f_i_* is the binary indicator vector for sequence *i*, where a value of 1 for *f_ik_* indicates that feature *k* is active for sequence *i*, and ⊗ is the vector product operator. Here, the value of κ has been added to the parameters of the Gamma distribution from which the values of η^(*i*)^_*jk*_ are drawn. This addition contributes some extra “stickiness” to the state transitions, putting more emphasis on intra-state vs. inter-state transitions (Fox et al., 2011).

We used the Bayesian nonparametric optimization process implemented in Michael Hughes' NPBayesHMM Matlab toolbox to fit the BP-HMM to our ensemble of data sequences (https://github.com/michaelchughes/NPBayesHMM). The toolbox employs Markov Chain Monte Carlo (MCMC) methods for learning and inference. The MCMC method alternates between resampling the binary feature assignments given observations and the current state of the dynamic properties of the model, and updating the properties of the model, given the binary feature assignments. An in-depth description of this process is beyond the scope of this report, but is described detail in Fox et al. (2009), Hughes et al. (2012).

##### BP-HMM parameter selection

The BP-HMM learning process requires specification of (nominally) 4 parameters used by the optimization routine. Each of these parameters influences the structure and properties of the final solution, and by tuning them appropriately, they can enforce domain-specific constraints on the final solution. Below, we describe the four parameters, their role in influencing the properties of the final model, the combination of values we experimented with, and the final set of parameters we selected:

- γ: The total number of active features in each data sequence has a Poisson distribution with mass parameter γ. Larger values of γ increase the number of features expected to be active in each sequence. We tested values of γ = {2, 3, 4, 5}.
- β: The beta process has a concentration parameter, β, which controls the degree to which features are shared across sequences. Larger values of β encourage more overlap in the set of active features across sequences. We set β = 1 in this analysis.
- κ: The state transition probabilities of the model govern the likelihood of transitioning from one state to the next, based on the current state. The “stickiness” parameter κ is used to place additional mass on the distribution governing self-transitions. This encourages discovery of a model that can generate state sequences with more temporal persistence, favoring longer intervals of intra-state occupation over frequent inter-state transitions (Fox et al., 2009). Larger values of κ create more intra-state “stickiness.” We tested values of κ = {50, 100, 200}.
- λ_0_: The observation probability distributions of each feature are modeled as categorical distributions. The optimization routine learns the parameters of these distributions using Bayesian non-parametric methods, which require specification of a conjugate prior for these distributions. The natural conjugate prior of a multinomial distribution is a uniform Dirichlet distribution, with concentration hyperparameter λ_0_. Smaller values of λ_0_ encourage sparser categorical distributions, while larger values nudge the distribution closer to uniform. The choice of λ_0_ also has an overall effect on the total number of features the modeling process generates. Conceptually, features with smoother distributions individually explain more of the data than those with more sparse distributions, thus requiring fewer total features to explain all of the data in the ensemble. We experimented with values of λ_0_ = {2, 3, 5, 10}.

We chose large values of κ to strongly encourage intra-state transitions. Any additional parameters were set to the default values implemented in the NPBayesHMM toolbox. For each parameter combination, we ran the optimization algorithm for 10,000 iterations, and selected the model parameters at the last iteration as the final set.

##### BP-HMM model selection

Each parameter combination generated a slightly different model, so we implemented a clustering technique to identify individual models that were representative of the unique properties of all the models in the ensemble. We first grouped the models by number of features, generating 7 groups with feature libraries of size *s* = {7, 8, 10, 11, 13, 14, 15}.

For groups with more than one member, we chose a single model to represent the group of solutions using the following method:

- For a group with feature library size *s*, we collected all of the 60-dimensional categorical observation probability distribution parameters from each member, and clustered them into *s* clusters using K-means with a Euclidean distance metric.
- For each group member, we computed the distance between each of its categorical distribution vectors and its assigned cluster centroid, and then summed these values, generating a metric representative of how close each distribution fell to the full set of cluster centroids.
- The solution with the minimum total distance to cluster centroids was selected as the representative solution of the *s*-state models.

Clustering the solutions in this way was designed to identify the single solution within each group that had categorical distribution parameters most similar to all of the other group members.

Although our final set of models generated 7 different representations of the data, we observed that each model was capturing essentially the same dynamics in each sequence with varying levels of granularity dictated by the number of states in the model. For the sake of simplicity, we have chosen to present our final analysis of the 10-state model only. We felt that this model represented the best trade-off between under- and over-fitting of the dataset. The parameter combination corresponding to the final 10-state model is {γ = 3; λ_0_ = 5; κ = 200; β = 1}.

#### Vector quantization

The data sequences to be modeled are not natively discrete, and therefore must be re-coded to satisfy the constraints of the discrete HMM representation. The 2-dimensional mixed state categorical/continuous feature vector derived from the players' activity logs were converted to discrete feature vectors using a simple vector quantization technique.

**Figure 4:**
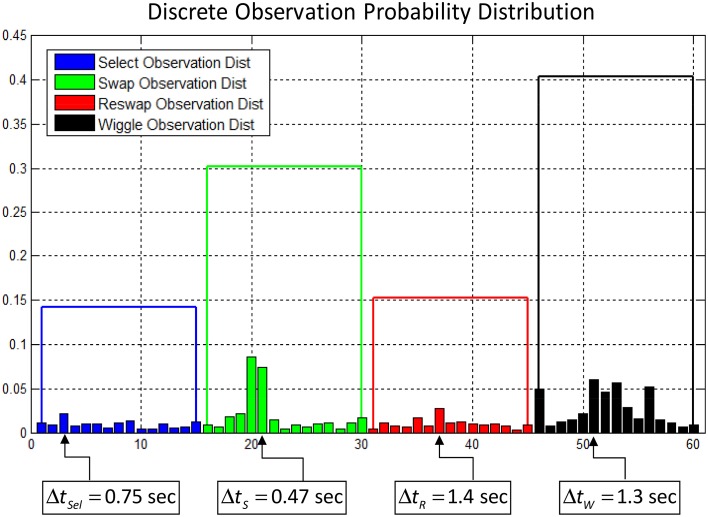
**Example of a discrete observation probability distribution of the 60-symbol alphabet used to encode the activity logs and temporal features**. Each small bar corresponds to a centroid representing a range of temporal inter-activity values. The height of the bar represents the relative probability of observing the corresponding symbol. The bars are grouped by activity log type, and the height of the larger blocks is equal to the total probability of observing each activity log, summing across all temporal values. The actual inter-activity times represented by one symbol from each group are depicted below the graph.

For each of the four activity log types (*select*, *swap*, *reswap*, and *wiggle*), all of the corresponding continuous-valued temporal features were clustered using K-means clustering. A set of centroids representing clusters of temporal values were identified, and the data points assigned to each cluster were re-coded with a corresponding cluster number. The K-means algorithm requires *a priori* specification of the number of clusters to generate from the dataset, and through experimentation we decided to use 15 clusters to represent the range of temporal features for each activity log. Each data sequence was re-coded using this vector quantization scheme, generating a new categorical observation vector composed of values from a 60-symbol alphabet, where each symbol represents both an activity log and temporal feature centroid. Figure 4 depicts a sample discrete observation probability distribution for the 60 possible observations. The height of each small bar corresponds to the probability of observing the symbol, and each bar corresponds to a centroid representing a cluster of inter-activity temporal feature values. The bars are sorted in order of increasing inter-activity time value (faster 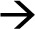 slower) from left to right for each activity type. The parameters describing each hidden state's observation probabilities characterize the behavior of the individual in that state in terms of what activities they performed and how quickly they performed them.

Using the procedures described above, we fit a BP-HMM to the ensemble of data collected in our pilot study and characterized the quantitative parameters of the model in terms of actual observed subject behaviors in the game-play context. We also used participants' questionnaire data and task performance metrics to quantitatively (statistically) examine whether the BP-HMM approach meaningfully captured true variation in participants' behavior. In this way, we attempted to provide robust evidence for the validity of BP-HMM approach as a method for analyzing sequential behavior.

## Results

### Overall task performance

As part of post-session questionnaires, participants were asked to provide, in their own words, what they believed the game-play rule was that governed the accumulation of points. These responses were then scored as “correct” or “incorrect” by two independent raters who were blind to participants' condition and BP-HMM modeling results. Participants who simply reiterated the instruction (hint) to “score points by grouping like objects” were not given “correct” ratings. After initial ratings were made, inter-rater reliability statistics were computed using Cohen's Kappa model (Cohen, 1960; Sim and Wright, 2005) for participants' responses following each session. Kappa statistics for sessions 1 and 2 were 0.71, falling well within an acceptable range of agreement given the number of observations and high a priori likelihood of agreement (50%) (Sim and Wright, 2005). Following estimation of inter-rater agreement, the singular discrepancy was reconciled. See **Table 2** for correct/incorrect determinations for session 1 reports, and **Table 3** for session 2 reports. Overall, response rates were poor. Of the participants who did report, those that correctly identified the rule (*n* = 5) were also those given an initial “hint.” However, many participants who were given this “hint” did not correctly identify the rule.

The number of *swaps* each participant made during the task is synonymous with points gained; they reflect successful groupings of tiles. Therefore, the number of *swaps* serves as a continuous performance metric as well. To characterize gross performance on this metric, we averaged the number of *swaps* exhibited by each participant across both sessions, as well as participants' Likert-Scale responses for both post-session reports of task difficulty, workload, and engagement. We examined differences on these three measures across the four conditions—Wiggle w/o Instructions, Static w/o Instructions, Wiggle w/Goal Instructions, and Static w/Goal Instructions. One participant was removed from all analyses due to systemic “floor effect” in post-session reports (gave all low scores, ignoring item wording). A simple One-Way Analysis of Variance (ANOVA) revealed no significant differences for either self-reported task engagement or task-related workload. However, we found that the average number of *swaps* is significantly different across conditions [*F*_(3, 18)_ = 10.66, *p* < 0.01], and that self-reports for task difficulty also significantly differed across conditions [*F*_(3, 16)_ = 8.90, *p* < 0.01]. *Post-hoc* comparisons (Bonferroni corrected) further revealed that for both significant effects, the Static w/Goal Instructions condition was significantly different from all other conditions. Participants in the Static w/Goal Instructions condition uniformly exhibited more *swaps* than any other condition (*p* = 0.01–0.001), and reported less task difficulty (*p*-value range = 0.09–0.002). Additionally, spot-checking of participant data revealed that these effects were not driven by any single outlier, but represent a uniform difference between conditions; participants in the Static w/Goal instructions condition ranged between an average of 134–336 swaps during their sessions, exceeding the average number of swaps in any other condition (see Table 1). This suggests that participants benefited from knowing that the goal of the game was to acquire points, and that the wiggle function added no value. In fact, without appropriate context (e.g., explicit instructions), the added functionality may have made the game more complicated for participants. These findings were further explored through both heuristic and quantitative decomposition of BP-HMM models.

**Table 1:** **Cell means and standard deviations for task-dependent performance and experience measures**.

**Independent variables × measure**	**Wiggle condition: wiggle function enabled**			**Static condition: wiggle function disabled**		
	**Mean swaps**	**Mean difficulty**	**Mean engaged**	**Mean swaps**	**Mean difficulty**	**Mean engaged**
No instruction condition: no instructions given	*M* = 33.33	*M* = 6.80	*M* = 3.00	*M* = 56.25	*M* = 5.63	*M* = 2.80
	*SD* = 16.55	*SD* = 0.76	*SD* = 0.34	*SD* = 23.21	*SD* = 1.49	*SD* = 0.34
Instruction condition: only given goal to acquire	*M* = 20.75	*M* = 6.67	*M* = 2.86	*M* = 207.40	*M* = 3.7	*M* = 2.85
the most points	*SD* = 17.73	*SD* = 1.04	*SD* = 0.73	*SD* = 109.41	*SD* = 0.84	*SD* = 0.60

*Means are values for each measure, averaged across both play session. Swaps indicate successful groupings of tiles in the Wiggle game; therefore, mean swaps are redundant with game-related performance. Self-reports for task-dependent difficulty and engagement were sampled following each session in Post-Session surveys*.

### BP-HMM modeling results

The BP-HMM parameters characterize subjects' interactions with the computer game. We interpreted these parameters in the context of the actual dynamics of game-play, and developed qualitative heuristics and quantitative metrics for identifying distinct patterns of strategic interactions with the game interface. We then evaluated these metrics against independently collected data from intake and post-session questionnaires. This approach was designed to determine if the models were able to systematically explain variation in the independent data, or if the BP-HMM method was merely “fitting noise,” providing no inferential value for understanding participants' experiences with the game. The analysis was by necessity *post-hoc*, since the models generated by the BP-HMM are completely data driven, making traditional a priori hypothesis-testing approaches to model validation impossible.

#### Shared feature activation patterns

**Figure 5:**
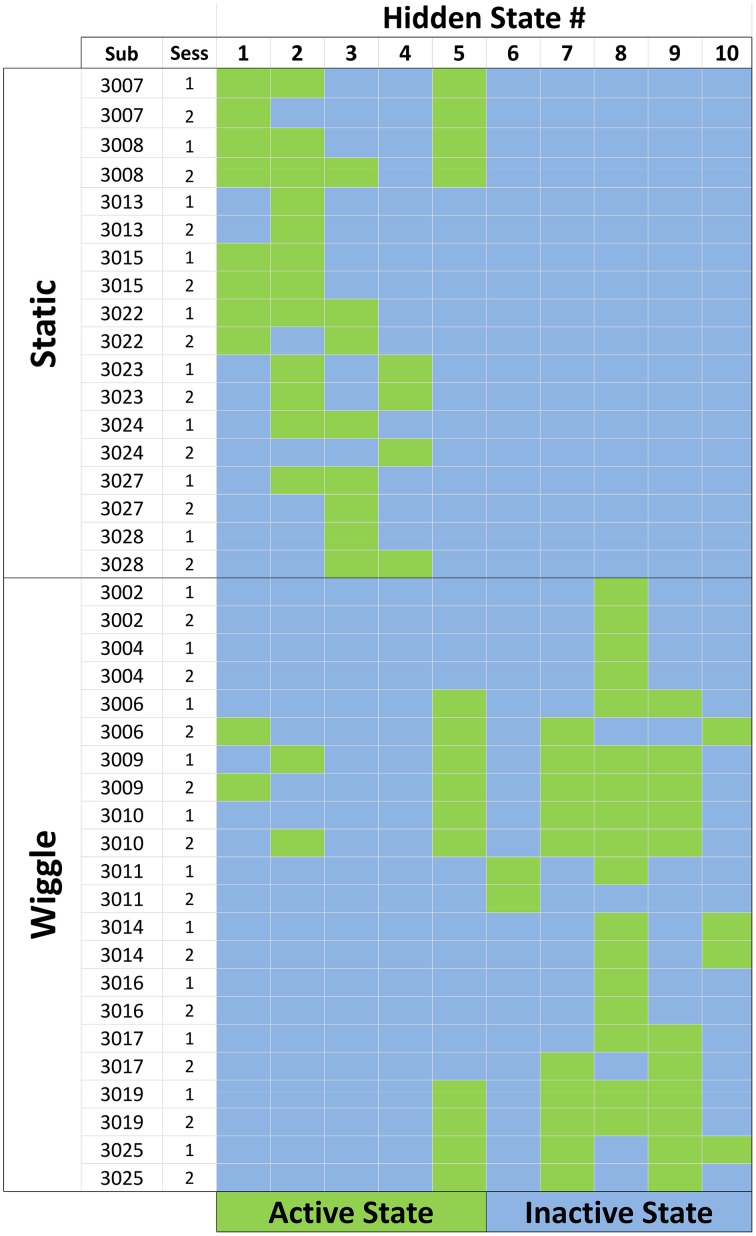
**Feature activation map for the ensemble of data sequences**. Each row corresponds to a single session for a participant. Static participants had the wiggle tile-sorting function disabled during their interactions with the game. Columns correspond to the number assigned to each hidden state, and green cells indicate that the state in that column was active for the corresponding participant/session.

Our subject population was separated into sub-groups of people with/without the wiggle function enabled in the game. By jointly modeling all sequences together, we were able to identify patterns of behavior exhibited by participants in *both* the static and wiggle conditions, as well as patterns that were unique to the experimental conditions. The ensemble feature activation map captures the shared feature structure, as shown in Figure 5. The rows of the map correspond to each data sequence, and they are sorted by subject condition, with all static participants clustered in the top rows, and wiggle participants toward the bottom. The columns of the matrix correspond to model states (behaviors). Green cells indicate “active” features in each sequence. The states were sorted according to the sum of the probability values assigned to wiggle-related symbols in each of the states, increasing from left to right (1 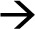 10). Sorting the features in this way reveals activation patterns clustered on the diagonal for the static and wiggle groups, indicating that behaviors unique to subject condition are captured by the properties of different states. Based on the total probabilities assigned to wiggle-related symbols, we observed that the model automatically created 5 states with a non-negligible probability of observing a *wiggle* move, and 5 with probabilities close to zero. We will refer to these groups of features as “wiggle states” (6 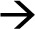 10) and “static states” (1 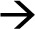 5), respectively. Theoretically, all wiggle-related symbols in the static states should have exactly zero probability of occurrence. However, the stability of the optimization routine used to model the ensemble requires that there be at least some small likelihood assigned to each symbol in the alphabet, resulting in the non-zero probabilities we observed. We felt that this small inaccuracy was acceptable given that it allowed us to model all sequences simultaneously, regardless of subject condition.

#### Heuristics extracted from hidden state distribution parameters

The observation probability distributions characterize subjects' interactions with the computer game in terms of what they did and how quickly they did it. Within the 10-state model, we heuristically identified subsets of behaviors representative of distinctly different strategies for naïve exploration of the game interface, and behaviors representative of mastery of the rules of the game.

##### Random search states

**Figure 6:**
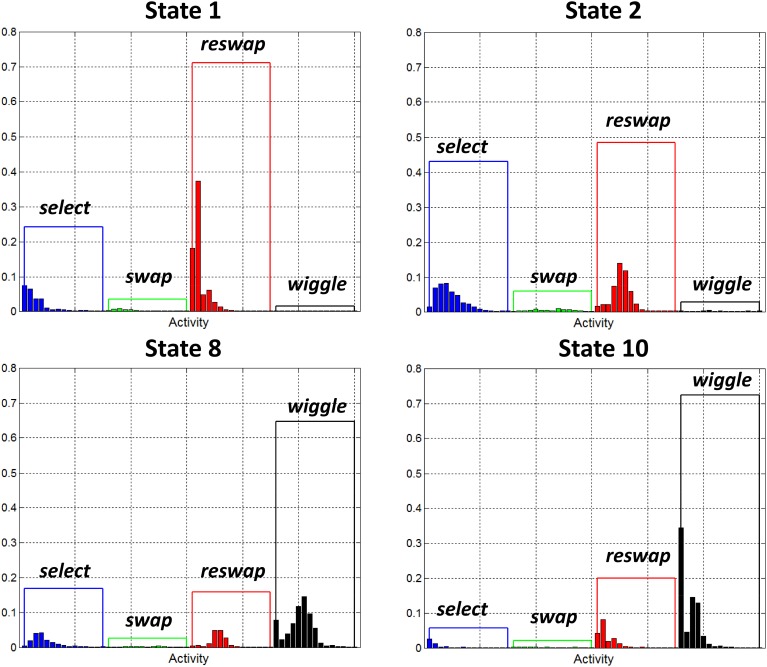
**Observation probability distributions for states 1, 2, 8, and 10**. Each of these states has been identified as a “random search” state. They represent the behavior of participants who haven't yet discovered how to score points in the game, as demonstrated by the low probabilities assigned to *swap* activities. Wiggle states 8 and 10 are dominated by *wiggle* activities, indicating that while in these states, participants spent most of their time exploring that function.

Figure 6 depicts the activity distributions for states 1, 2, 8, and 10. The distributions of the static states (1 and 2) are dominated by *swap* and *reswap* activities. The relationship between these activities captures the degree to which the participants moved around the full grid of tiles as they attempted to identify the color matching rule that scores points. The negligible number of *swaps* and large number of *reswaps* indicates that individuals observed in these states haven't yet discovered the color matching rule—they are still experimenting. The dominant, co-occurrence of *select* and *reswap* activities in these states suggests an element of random exploration. The relatively large probabilities of *select* activities indicates that participants observed in these states were searching for patterns across the entire set of tiles, rather than focusing on a particular sub region. A similar pattern emerged in state 8, with the addition of *wiggle* activities. We also categorized wiggle states 9 and 10 as random search behaviors, although they are dominated more heavily by *wiggle* activities.

##### Sequential search states

**Figure 7:**
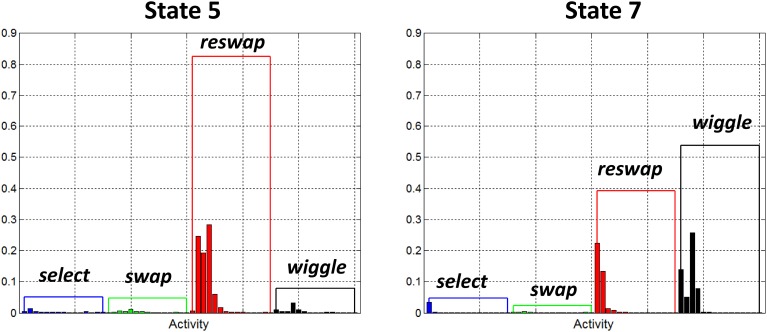
**Observation probability distributions for states 5 and 7**. Each of these states has been identified as a “sequential search” state. They represent behavior of participants who haven't yet discovered how to score points in the game, as demonstrated by the low probabilities assigned to *swap* activities. We believe these states are capturing a specific type of search strategy wherein participants systematically, sequentially selected adjacent tiles moving across rows and down columns.

Figure 7 depicts the activity distributions of states 5 and 7 which are dominated by *reswap* activities in static state 5, and *reswap* + *wiggle* activities for wiggle state 7. As in the random search states, the negligible probability assigned to *swap* activities in these states indicates that they capture behavior of individuals who haven't yet discovered the rule. However, the probabilities associated with *select* symbols are also very low, indicating that new tile selections were limited to the region directly adjacent to the currently highlighted tile. The properties of this state appear to capture a behavior pattern we observed, which can qualitatively be described as a “sequential search” strategy. Participants exhibiting this behavior systematically, sequentially selected adjacent blocks horizontally across rows, and vertically down columns. Participants with the wiggle function enabled also exhibited this behavior, interleaved with periods of *wiggle* activity.

##### Success states

**Figure 8:**
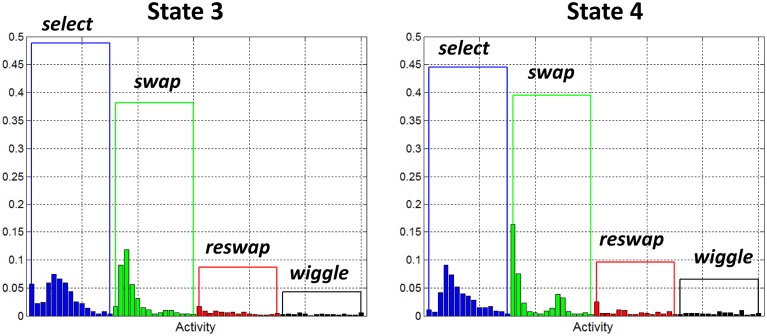
**Observation probability distributions for states 3 and 4**. Each of these states has been identified as a “success” state. They represent behavior of participants who have discovered the rules of the game, as demonstrated by the large probability assigned to the *swap* activities, and relatively small probabilities assigned to *reswaps*.

Figure 8 depicts the activity distributions for states 3 and 4, which are dominated by *select* and *swap* activities in the static states (3 and 4), with a negligible contribution from *reswaps*. Of all the wiggle-states, state 6 was the only one with a non-negligible contribution from *swap* activities, so it appears as though it is a success state, or at least more successful than search states.

#### State sequence statistics

The BP-HMM assigns a subset of states to each sequence from the shared global library, and identifies unique state transition probabilities for each sequence. From these model elements, we identified the most likely state sequences from each raw activity sequence. We then calculated the amount of time spent in each state by each participant in each session. The state sequence and relative amount of time spent in each state capture the dynamics of participants' strategies as they evolve across each session of game play. When paired with performance criteria, this information yielded insights into which strategies/combinations of strategies led to successful identification of the rules of the game, and how knowledge condition and access to the wiggle function affected strategy and performance.

**Table 4** contains the proportion of time spent in each state by each participant for each session, and whether they were “successful” in discovery of the game rule from the perspective of modeling output, based solely on the criteria that they spent a non-negligible proportion of time in the states exhibiting high *swap* rates (3, 4, 6). Analysis of the state-duration data based on this heuristic revealed the following observations:

First, “goal” condition participants were more likely to discover the rule. The only participants who discovered the rules of the game were those who were told at the beginning of their session that the goal was to “get points by grouping like objects.” All of the static (non-wiggle) participants who were given this hint eventually discovered the rule. The only participant in the wiggle condition who discovered the rule was also given this hint. Second, access to the wiggle function did not seem to aid in the discovery of the rules of the game. Only one of the 11 participants with wiggle functionality spent time in any of the success states. In all of the wiggle states, the probabilities associated with *wiggle* activities dominated all others, indicating that these participants actually spent most of their time wiggling, regardless of state, to the detriment of discovery of the rules of the game.

Finally, sequential search strategies did not appear to aid in rule discovery. No participants who spent any time in the “sequential-search states” (5 and 7) were successful. For the static participants, none of the individuals who were given the rule hint employed the sequential search strategy at all. The raw activity rates observed during sequential search intervals tended to be much higher than those recorded during random search. This method inevitably generated random matches, simply due to the physics of the game, but the agnostic selection of tiles in a deterministic pattern may have made it more difficult to recreate the moves that generated these random matches, which could aid in rule discovery.

#### Comparisons between heuristic decomposition of modeling output and independent performance metrics

A comparison between independent raters' assessment of the participants' understanding of the rules for scoring points in the game, and those identified through heuristic interpretation of the modeling parameters reveals substantial overlap. Of the 5 participants identified by raters as discovering the rule after session 2 (see Table 3), all of them were similarly identified by model-driven heuristics (see Table 4). One participant that was not classified as successful by the raters in session 1 (see Table 2) was identified through model-driven heuristics, although the same participant was identified as having successfully identified the rule in session 2 by both methods. One additional participant who was classified as successful from model-based criteria gave no data in post-session reports. While quantitative performance metrics (e.g., n *swaps*) clearly align with heuristic assessments of BP-HMM modeling output, because swapping behavior was used to train the models, it cannot be thought of as an independent metric, so comparative findings are not informative.

**Table 2:** **Verbatim self-reported rule descriptions following Session 1 of game play, by subject**.

**Subject**	**Condition**	**Rule description**	**Correct?**
1	Wiggle No hint	N/A	
2	Wiggle Hint	Moving the cross	No
3	Wiggle Hint	I think there was no rule and it colors moved automatically	No
4	No wiggle No hint	N/A	
5	No wiggle No hint	Don't think just act…	No
6	Wiggle No hint	Touching different squares	No
7	Wiggle No hint	Choosing certain colors	No
8	Wiggle No hint	N/A	
9	No wiggle No hint	N/A	
10	Wiggle Hint	Just clicking on one particular color box	No
11	No wiggle No hint	There was no rule	No
12	Wiggle No hint	N/A	
13	Wiggle No hint	Not sure but I think it was about getting a certain color combination	No
14	Wiggle No hint	To put colored squares into groups and clusters	No
15	No wiggle Hint	Finding and clicking a single color squares surrounded by three squares of a different color	No
16	No wiggle No hint	Match three or more colors in a group next to each other	Yes
17	No wiggle Hint	Three similar colored blocks needed to align, two blocks together with one block on top/side of the other two blocks	Yes
18	Wiggle No hint	The rule was to be fast, because gaining points was totally random	No
19	No wiggle Hint	Acquire points by grouping 3 or more boxes of the same color together	Yes
20	No wiggle Hint	Move one block to make at least 3 touching/adjacent blocks of the same color	Yes

*N/A indicates that participant gave no response*.

**Table 3:** **Verbatim self-reported rule descriptions following Session 2 of game play, by subject**.

**Subject**	**Condition**	**Rule description**	**Correct?**
1	Wiggle No hint	N/A	
2	Wiggle Hint	By grouping blocks with similar colors	No
3	Wiggle Hint	At first I thought I was making the colors move, but in the end i was convinced that it was automatic yet I still sought for the rule of the game	No
4	No wiggle No hint	N/A	
5	No wiggle No hint	Don't think too much…	No
6	Wiggle No hint	Touching the different squares	No
7	Wiggle No hint	N/A	
8	Wiggle No hint	N/A	
9	No wiggle No hint	N/A	
10	Wiggle Hint	Don't know the exact rule	No
11	No wiggle No hint	There is no rule	No
12	Wiggle No hint	N/A	
13	Wiggle No hint	The more of the same colors the higher the score	No
14	Wiggle No hint	To put colored squares into groups and cluster them	No
15	No wiggle Hint	Turn three squares of the same color into four squares by annexing a fourth neighboring square of a different color	Yes
16	No wiggle No hint	Still matching 3 or more of the same color	Yes
17	No wiggle Hint	Three matching colored blocks needed to line up, in any direction	Yes
18	Wiggle No hint	Gain more points by moving methodically across the screen/row	No
19	No wiggle Hint	Points are acquired by moving colored blocks one space at a time to construct groups of 3 or more boxes of the same color	Yes
20	No wiggle Hint	Move one block to make sure 3 adjacent blocks of the same color are touching	Yes

*N/A indicates that participant gave no response*.

**Table 4:** **Proportion of session time each participant spent in each state**.

**Subject**	**Session**	**Hint?**	**Hidden state #**									
			**1**	**2**	**3**	**4**	**5**	**6**	**7**	**8**	**9**	**10**
**STATIC**												
4	1	N	0.67	0.29			0.04					
4	2	N	0.86				0.14					
5	1	N	0.22	0.13			0.65					
5	2	N	0.31	0.42	0.04		0.23					
9	1	N		1								
9	2	N		1								
11	1	N	0.25	0.75								
11	2	N	0.91	0.09								
15	1	Y	0.25	0.32	0.43							
15	2	Y	0.40		0.60							
16	1	Y		0.12		0.88						
16	2	Y		0.05		0.95						
17	1	Y		0.88	0.12							
17	2	Y				1						
19	1	Y		0.04	0.96							
19	2	Y			1							
20	1	Y			1							
20	2	Y			0.30	0.70						
**WIGGLE**												
1	1	N								1		
1	2	N								1		
2	1	Y								1		
2	2	Y								1		
3	1	Y					0.01			0.95	0.04	
3	2	Y	0.03				0.01		0.22			0.74
6	1	N		0.09			0.17		0.01	0.69	0.04	
6	2	N	0.17				0.39		0.16	0.05	0.23	
7	1	N					0.01		0.09	0.81	0.09	
7	2	N		0.02			0.31		0.04	0.56	0.08	
8	1	Y						0.65		0.35		
8	2	Y						1				
10	1	Y								0.66		0.34
10	2	Y								0.07		0.93
12	1	Y								1		
12	2	Y								1		
13	1	N								0.14	0.86	
13	2	N							0.07		0.93	
14	1	Y					0.08		0.15	0.11	0.66	
14	2	Y					0.01		0.01	0.50	0.48	
18	1	N					0.01		0.37		0.10	0.52
18	2	N					0.11		0.82		0.07	

*Participants who eventually discovered the color-matching rule are highlighted green. Participants in the “goal” condition—told to “group like-colored tiles”—are highlighted in blue*.

### Statistical assessment of shared information between modeling output, game performance, and independent self-report data

The purpose of a heuristic decomposition of the BP-HMM model parameters as they relate to performance metrics serves to illustrate the interpretability and qualitative insight that the approach yields. We also examined whether quantitative metrics extracted from the model contain information that is stochastically related to independent data from intake and post-session questionnaires, and assessed the extent to which BP-HMM modeling outputs add information beyond raw data extracted from game play, such as activity rate. The goal of these analyses was to evaluate whether the approach produces information that is meaningfully related to user experience and performance, or whether it is simply modeling noise.

#### Derivative metrics from modeling output

The BP-HMM states encode the temporal relationships between activities performed by participants interacting with the software. Therefore, a key comparison to be made is how the shape of the distributions describing these relationships differs between the states. States with platykurtic (diffuse) distributions represent modes of behavior that reflect integration of the available software functionality (a uniform probability of observing activities within a state). States with a leptokurtic (peaked) distribution represent models of behavior reflecting a strong behavioral bias for specific activities. By classifying each state as having a peaked or diffuse distribution and combining information from state sequence activities, one can then quantitatively describe each participants' behavior based on the percentage of time they spent in states with either peaked or diffuse distributions. This kind of metric putatively captures the participants' understanding for how to integrate the activities to accomplish the task, rather than use them independently.

Using this “peakedness” metric, we sorted the states into two groups in order to maximize power in analyses with self-reports. We experimented with two different methods for grouping the states, in an effort to identify a robust solution (see Table 5 for classification results). First, we collapsed the 60-dimensional categorical distributions into 4 dimensions by summing the probabilities assigned to the 15 centroids (see small bars Figure 4) representing each of the 4 activity types. We then estimated the population excess kurtosis of the four values for each state, and classified states as “diffuse” (platykurtic or uniform) or “peaked” (leptokurtic) based on whether values reached excess kurtosis for uniform distributions (−1.2; Decarlo, 1997).

**Table 5:** **Classification of states based on kurtotic qualities of categorical distributions**.

**States**		**Kurtosis of average (−1.2)**	**Average of maximum (0.039)**
State 1	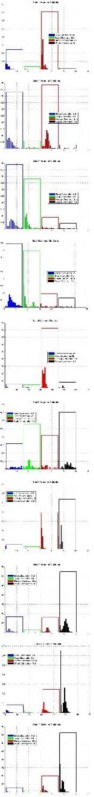	1.714	0.047
State 2		−5.652	0.032
State 3		−4.657	0.033
State 4		−5.568	0.030
State 5		3.973	0.055
State 6		−1.402	0.023
State 7		−4.393	0.036
State 8		3.104	0.043
State 9		1.714	0.044
State 10		2.771	0.048

*Values indicate metrics describing the peakedness of categorical state distributions from centroid values. Parentheses indicate cutoff points for classification—excess values are classified as peaked. Red colored cells are classified as peaked states, blue as diffuse states*.

Second, we took the average of the centroid values in each activity, for each state, and interpreted the maximum of these 4 values as a measure of “peakedness” of that state. Using the average of these values across all states, we categorized states as “peaked” if their state-specific average was above the maximum average value across all states, and as “diffuse” if it fell below.

Both classification methods resulted in the same decisions. States 1, 5, 8, 9, and 10 fell into the “peaked” category, and states 2, 3, 4, 6, and 7 fell into the “diffuse” category. We then calculated the percentage of game time that participants were observed in the peaked category of states, for each session, and across both sessions. Given that these variables were calculated as percentages, subtracting 1 from these values would give the percentage of time participants were observed in the diffuse category of states.

#### Shared information between modeling output and independent self-reports

**Figure 9:**
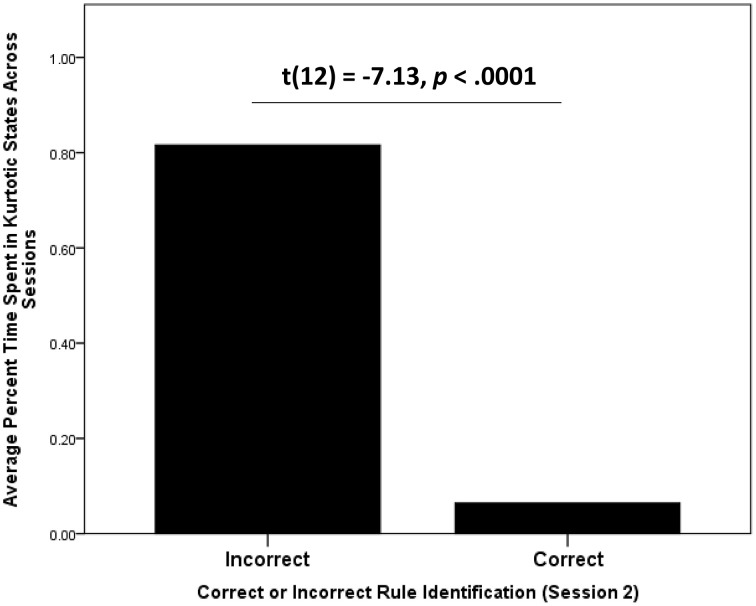
**Average time spent in kurtotic states by participants who were identified by blind raters as having correctly described the rule governing how to accumulate points in Wiggle**. Participants who correctly reported the rule after both sessions were significantly less likely to spent time in kurtotic BP-HMM states.

**Figure 10:**
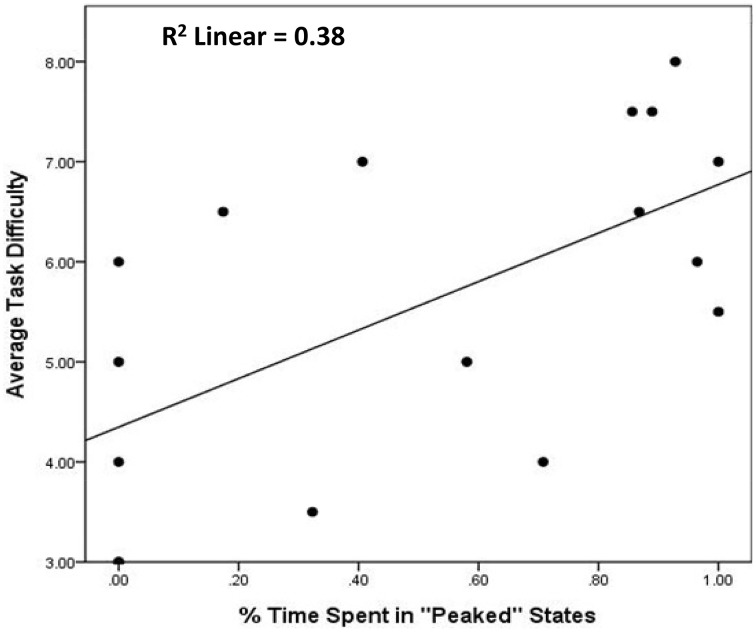
**Association between average time spent in peaked states and self-reported task difficulty**. Illustrates that BP-HMM modeling output can be reduced to metrics with intuitive and predictive utility. First, metrics describing the structure of states have bearing on users' understanding of how game interface functions are integrated. Second, participants who rely on specific functions, without exploring (or trying to discover) other ways of interacting with the game interface report difficulty with the game task (above).

Correlation analyses revealed a strong relationship between the time participants spent in peaked and diffuse states and game performance across sessions. Participants who spent more time in peaked states than diffuse states were less likely to correctly swap tiles in their first session (*r* = −0.68, *p* < 0.01). This is to be expected given that the states that exhibited more swapping behavior were agnostically classified as diffuse states. However, almost all of the participants who spent more time in peaked states were almost categorically incorrect in post-session 2 self-reports of the governing rule for scoring points, as indicated by independent raters [*t*_(12)_ = −7.13, *p* < 0.001; see Figure 9]. They also self-reported expending more mental effort in their first session (*r* = 0.49, *p* < 0.05), but not their second (*r* = 0.25, *p* = 0.05), and were more likely to report more difficulty with the task across sessions (*r* = 0.62, *p* < 0.01, see Figure 10).

We also found that participants who spent more time in peaked states exhibited a higher activity rate (*r* = 0.49, *p* < 0.05), generating more logs across sessions. Moreover, high activity rates across sessions were related to incorrect self-reports of the Wiggle game rule [*t*_(12)_ = −3.06, *p* < 0.01] and self-reported task difficulty across sessions (*r* = 0.57, *p* < 0.05). Therefore, we examined whether the BP-HMM derived peakedness metric was a stronger indicator of incorrect self-reports, independent of activity rate in predicting self-reported task difficulty. Because our sample size was too limited to perform a step-wise logistic regression comparing the independent effects of time spent in peaked states and activity rate on incorrect self-reports of the rule in the same model, we calculated the effect sizes (Cohen's d) of each predictor from *t*-tests The effect of time spend in on correct/incorrect determinations was *d* = 4.11, a medium to strong effect size. However, the effect of activity rate was 1.77, a weak effect. To evaluate the independence of the two predictors on task difficulty we used step-wise regression. At the first step, we entered activity rate as a predictor of task difficulty. At the next, we added to the model the time spent in peaked states across sessions. We found that at the second step, activity rate is no longer a significant predictor of task difficulty (b^*^ = 0.35, *p* = 0.14), while the time spent in peaked states, remains a (marginally) significant predictor of task difficulty (b^*^ = 0.45, *p* = 0.07). Taken together, this suggests BP-HMM modeling output not only predicts independent performance and self-reports of experience in systematic ways, it also adds more predictive information than raw behavior alone. These findings generally align with insights from heuristic evaluations as well (above).

We also found that the correlation between the time spent in peaked states in session 1 is highly correlated with time spent in those states in session 2 (*r* = 0.84, *p* < 0.001). To investigate this further, we extracted each participant's unique state transition probabilities, and calculated the average likelihood that each participant would transition between diffuse and peaked states, and whether they would transition to other states within the same category (we excluded probabilities for transitioning into the exact same states as these tend to be much higher). A simple within-subject *t*-test indicates that participants were more likely to transition to states within the same category than to transition between categories of states [*t*_(18)_ = 6.01, *p* < 0.001]. These findings suggest that once participants discovered a suitable strategy for how to play the game, they generally didn't deviate from that strategy, even if it required more effort and resulted in fewer points scored.

We also conducted a One-Way ANOVA across the four experiment conditions—Static w/o Instructions, Static w/Goal Instructions, Wiggle w/o Instructions, Wiggle w/Goal Instructions—with the time spent in peaked states as a dependent variable, followed by corrected post-hoc pairwise comparison. Results show a main effect of condition [*F*_(3, 17)_ = 7.84, *p* < 0.01], with *post-hoc* comparisons illustrating that participants in the Static w/Goal condition evidenced less time spent in peaked states compared to Wiggle w/o Instruction (Δ*M* = −0.66, *p* < 0.05), and Wiggle w/Instruction Instructions (Δ*M* = −0.90, *p* < 0.01), but no difference from participants in the Static w/o Instruction (Δ*M* = −0.66, *p* = 0.15) category. This resonates with our comparisons against task performance—participants spending the most time in diffuse states also performed the best, and participants who performed the best were in the static rule condition. However, this analysis shows a demarcation between Static and Wiggle conditions in terms of how users integrated software functionality. It suggests that the insertion of a new activity may have led users to focus on wiggling over finding out how *select* and *swap* activities were related.

We did not observe any associations between the time spent in either diffuse or peaked states and intake self-reports. This was also true for raw behavioral data. However, we found that participants' likelihoods for transitioning from peaked states to other peaked states was inversely related to intake self-reports measuring a Need for Cognition (*r* = −0.75, *p* < 0.01) and positively related to self-reported Need for Closure (*r* = 0.55, *p* < 0.05). The Need for Cognition inventory taps proclivities related to pursuing mentally engaging, challenging problems with a reasoned approach. They are also related to success in analytic tasks and analytical aptitude (Cacioppo et al., 1984; Poore et al., 2014). In contrast, the Need for Closure scale is designed to capture a desire for unambiguous tasks and completion of problems for the sake of closure, rather than accuracy or completeness (Roets and Van Hiel, 2011; Poore et al., 2014). Taken together, these analyses suggest that BP-HMM models *can* capture meaningful information related to user experience and that this information can be informative beyond raw data extracted from software logs (e.g., activity rate).

## Discussion

In this methods report, we describe a novel implementation of the BP-HMM for modeling and analyzing behavioral data extracted from software activity logs. We conducted a pilot experiment using a simple game that required participants to learn the rules of that game (i.e., how to get points) through experimentation with its interface functionality. Some participants were given more information than others, and some participants were given more functionality to make sense of. After modeling activity logs generated during game-play using a BP-HMM, we identified systematic differences in the strategies participants used, based on the parameters of the model. Only a few participants demonstrated knowledge of the actual rules of the game, while others came close—they tended to occupy a few states that evidenced some degree of proficiency in integrating tool functions. Other participants' behavior appeared to indicate hypothesis testing and other exploratory patterns. Still others settled on sequential search patterns that generated some points, but were more effort-intensive.

Subsequent statistical analysis of raw performance indices (number of *swaps*) across the task design suggested that users with more information and less functionality to explore performed better, in general. Additional analysis of metrics derived from modeling output supported descriptive analyses. Quantitative assessment of the “peakedness” of the categorical distributions of each hidden state revealed two well-defined categories of states, representative of how diffuse or focused participants' interactions with the game's functions were. We find that the time users spent in these two categories of states was significantly related to participants' self-reports of task difficulty and expenditure of mental effort. Metrics derived from the model parameters also added value in predicting these outcomes beyond raw behavior (e.g., activity rate). Finally, we find that the likelihoods associated with participants' transitions to states within the same category were strongly and inversely related to individual difference measures of rational problem solving styles and desires for intellectual challenge. Taken together, we provide sufficient evidence that the BP-HMM approach for modeling behavior within software environments is capturing real variation in user behavior that relates to both task-dependent outcomes and individual difference measures.

The BP-HMM approach shows promise in enabling a wide range of discoveries from how people interact with software environments. For example, recent research in the cognitive neurosciences has focused on the rewarding aspects of gathering or “foraging” for information to reduce outcome uncertainty in both social and non-social environments (Behrens et al., 2008; Niv and Chan, 2011; Kolling et al., 2012; O'reilly, 2013; Chau et al., 2014). Much of this research is conducted with impoverished stimuli—static images presented in sequence. BP-HMM and other approaches for modeling behavior in dynamic virtual environments can provide rich information to support more complex modeling of the brain behavior-interactions that occur when individuals forage for information in more realistic task environments.

Our approach also has applications for intuitive software design, software adaptation, and personalized learning. The BP-HMM approach creates a progressive library of software usage states that might be leveraged to automatically indicate when users are exhibiting specific kinds of behavior that have previously been linked to positive or negative outcomes. For instance, in the simple computer game described in this study, we found that the sequential search strategy was not helpful in discovery of the rules of the game. In fact, none of the participants employing this strategy were able to figure it out. As part of an adaptive interface design, real-time user behavior can be monitored for patterns reminiscent of this strategy. If detected, feedback to the user, implicit or explicit, might then be provided to help steer them toward a more preferred state.

### Caveats

Our pilot validation study is very simple—additional replication and research is needed to ensure that the BP-HMM approach and derivative metrics are extensible to larger applications, with fewer intrinsic dependencies between functions. Modeling activity data to make inferences about performance, where one of those activities is convoluted with performance itself is also problematic. In addition, we did not collect any information from the participants regarding prior experience with similar computer games, so it is unclear how this factor may have influenced their interactions with the software. While our assessment of the validity of the BP-HMM approach was by necessity post-hoc, we believe we were able to identify many content agnostic metrics that revealed non-trivial associations with self-report data, and other behavioral data. This would advocate for cautious optimism in the extensibility of the BP-HMM approach.

Nonetheless, the Wiggle game provides a compelling test case, given that it is a simple task, with rules that are not unlike many popular commercial titles, such as Bejeweled or Candy Crush. Additionally, it provided an opportunity to scale the combinatorics of functionality by toggling the wiggle function for some users. The fact that users performed more poorly with the function activated is interesting given that the ability to grab like-colored elements on the screen to better see patterns in the tiles is highly effective. But, without proper context, the wiggle function only added to the burdens on participants to learn how it could be integrated with other functions, and how it could be used to score points.

### Future directions

Future efforts will examine the utility of BP-HMMs for the assessment of significantly more complex, vertically integrated data analysis tools designed to enable visualization and analysis of “Big Data.” A richer software environment provides significantly more opportunity to study how software integration affects naïve users' intuitive approaches to problem solving. The functionality and organization of the analytic should enable the problem solving process, but establishing a metric against which this can be judged is challenging. To that end, we will use the BP-HMM to identify patterns representative of analytic work flow, and examine whether the software functions impede or enable this flow, as measured by overall performance in an analytic task. We believe that this new work will help further extend these methods to fully immersive environments with user-independent behaviors, and enable behavioral scientists to make full use of software as a naturalistic human ecology that is also a measurement medium.

## Funding

This work was funded under Draper Internal Research and Development funding. Draper Laboratory has filed a provisional patent application relevant to this work, entitled “A method for inferring standardized human-computer interface usage strategies from software instrumentation and dynamic probabilistic modeling.”

### Conflict of interest statement

The author declares that the research was conducted in the absence of any commercial or financial relationships that could be construed as a potential conflict of interest.
