# Supplementary material for: Modeling strategic use of human computer interfaces with novel hidden Markov models
Source: Front Psychol. 2015 Jul 3;6:919. doi: 10.3389/fpsyg.2015.00919 (PMC4490801; doi:10.3389/fpsyg.2015.00919)
Supplement: Supplementary file 1 [file Table1.DOCX]

***Supplementary Material***

**Modeling Strategic Use of Human Computer Interfaces with Novel Hidden Markov Models**

**Laura J. Mariano^1^*, Joshua C. Poore^1^, David M. Krum^2^, Jana L. Schwartz^1^, William D. Coskren^1^, Eric M. Jones^1^**

^1^The Charles Stark Draper Laboratory, Inc., Cambridge, MA, USA

^2^University of Southern California, Institute for Creative Technologies, Playa Vista, CA, USA

*** Correspondence:** Laura J. Mariano, The Charles Stark Draper Laboratory, 555 Technology Square, Cambridge, MA, 02139, USA.

[lmariano@draper.com](mailto:lmariano@draper.com)

Table S1

Descriptive Statistics and Composite Scale Reliabilities for Variables Used

| Individual Difference Composite Variables | N | | Scaling | | Mean | | SD | Alpha  (Scale) |
| --- | --- | --- | --- | --- | --- | --- | --- | --- |
| Analytic Problems | 17 | 1 – 6 | | 2.79 | | 1.74 | | -- |
| Subjective Numeracy | 17 | 1 – 7 | | 4.53 | | 1.10 | | .95 |
| Cog. Reflections Test | 16 | 1 – 3 | | .94 | | 1.12 | | -- |
| Need for Cognition (NFC) | 16 | 1 – 7 | | 4.75 | | .96 | | .94 |
| Need For Closure (NFCL) | 16 | 1 – 7 | | 3.94 | | .87 | | .90 |
| Experiential Cog. Style (REI) | 16 | 1 – 7 | | 4.91 | | .86 | | .88 |
| Rational Cog. Style (REI) | 16 | 1 – 7 | | 5.19 | | 1.12 | | .96 |
| Maximization Scale | 16 | 1 – 7 | | 4.29 | | .93 | | .79 |
| Enjoyment Session 1* | 17 | 1 – 5 | | 3.29 | | .96 | | -- |
| Enjoyment Session 2* | 16 | 1 – 5 | | 3.44 | | 1.09 | | -- |
| Engagement Session 1* | 17 | 1 – 5 | | 2.77 | | .43 | | .65 |
| Engagement Session 2* | 16 | 1 – 5 | | 3.03 | | .63 | | .77 |
| Task Difficulty Session 1* | 17 | 1 – 10 | | 5.53 | | 1.33 | | -- |
| Task Difficulty Session 2* | 16 | 1 – 10 | | 5.50 | | 2.10 | | -- |
| Task Effort Session 1* | 17 | 1 – 10 | | 5.06 | | 1.64 | | -- |
| Task Effort Session 2* | 16 | 1 – 10 | | 5.63 | | 2.22 | | -- |
| Activity Rate (/min) Session 1† | 19 | 1 – 10 | | 72.00 | | 61.18 | | -- |
| Activity Rate (/min) Session 1† | 19 | Cont. | | 96.88 | | 78.91 | | -- |
| N Swaps Session 1† | 19 | Cont. | | 33.16 | | 44.37 | | -- |
| N Swaps Session 2† | 19 | Cont. | | 48.16 | | 55.41 | | -- |
| N Transitions Session 1† | 19 | Cont. | | 12.53 | | 16.93 | | -- |
| N Transitions Session 2† | 19 | Cont. | | 20.42 | | 28.53 | | -- |

Note: Coefficient Alpha calculated from Cronbach model. Blank cells indicate that reliabilities cannot be calculated for a given variable (e.g., single item, number correct items). * Indicates data taken from post-task surveys, † indicates task data. Cont. is a continuously scaled measure.
