# Supplementary material for: Modeling strategic use of human computer interfaces with novel hidden Markov models
Source: Front Psychol. 2015 Jul 3;6:919. doi: 10.3389/fpsyg.2015.00919 (PMC4490801; doi:10.3389/fpsyg.2015.00919)
Supplement: Supplementary file 2 [file Table2.DOCX]

***Supplementary Material***

**Modeling Strategic Use of Human Computer Interfaces with Novel Hidden Markov Models**

**Laura J. Mariano^1^*, Joshua C. Poore^1^, David M. Krum^2^, Jana L. Schwartz^1^, William D. Coskren^1^, Eric M. Jones^1^**

^1^The Charles Stark Draper Laboratory, Inc., Cambridge, MA, USA

^2^University of Southern California, Institute for Creative Technologies, Playa Vista, CA, USA

*** Correspondence:** Laura J. Mariano, The Charles Stark Draper Laboratory, 555 Technology Square, Cambridge, MA, 02139, USA.

[lmariano@draper.com](mailto:lmariano@draper.com)

Table S2

Cross Correlations Between Intake Measures

| Intake  Self-Report  Measures | Analytic Problems | Subjective Numeracy | Cog. Reflections Test | Need for Cognition (NFC) | Need For Closure (NFCL) | Experiential Cog. Style (REI) | Rational Cog. Style (REI) | Maximization Scale |
| --- | --- | --- | --- | --- | --- | --- | --- | --- |
| Analytic Problems | 1.00 | -0.33 | 0.24 | -0.03 | -0.17 | -0.31 | 0.00 | -0.08 |
| Subjective Numeracy | -0.33 | 1.00 | 0.31 | .62^*^ | -0.09 | 0.25 | .53^*^ | 0.29 |
| Cog. Reflections Test | 0.24 | 0.31 | 1.00 | 0.45 | -0.24 | 0.08 | 0.37 | -0.01 |
| Need for Cognition (NFC) | -0.03 | .62^*^ | 0.45 | 1.00 | -.50^*^ | 0.29 | .94^***^ | 0.21 |
| Need For Closure (NFCL) | -0.17 | -0.09 | -0.24 | -.50^*^ | 1.00 | 0.20 | -.60^*^ | 0.20 |
| Experiential Cog. Style (REI) | -0.31 | 0.25 | 0.08 | 0.29 | 0.20 | 1.00 | 0.04 | 0.32 |
| Rational Cog. Style (REI) | 0.00 | .53^*^ | 0.37 | .94^**^ | -.61^*^ | 0.04 | 1.00 | 0.15 |
| Maximization Scale | -0.08 | 0.29 | -0.01 | 0.21 | 0.20 | 0.32 | 0.15 | 1.00 |

Note: * = *p* < .05; ** = *p* < .01; *** = *p* < .001.
