# Supplementary material for: Modeling strategic use of human computer interfaces with novel hidden Markov models
Source: Front Psychol. 2015 Jul 3;6:919. doi: 10.3389/fpsyg.2015.00919 (PMC4490801; doi:10.3389/fpsyg.2015.00919)
Supplement: Supplementary file 3 [file Table3.DOCX]

***Supplementary Material***

**Modeling Strategic Use of Human Computer Interfaces with Novel Hidden Markov Models**

**Laura J. Mariano^1^*, Joshua C. Poore^1^, David M. Krum^2^, Jana L. Schwartz^1^, William D. Coskren^1^, Eric M. Jones^1^**

^1^The Charles Stark Draper Laboratory, Inc., Cambridge, MA, USA

^2^University of Southern California, Institute for Creative Technologies, Playa Vista, CA, USA

*** Correspondence:** Laura J. Mariano, The Charles Stark Draper Laboratory, 555 Technology Square, Cambridge, MA, 02139, USA.

[lmariano@draper.com](mailto:lmariano@draper.com)

Table S3

Cross Correlations Between Session 1 and Session 2 Task-Related Measures

| Task-Related  Measures | Activity Rate (/min) (2) | N Swaps  (2) | N Transitions  (2) | % Time in  Peaked  States  (2) | Enjoy-  ment†  (2) | Engage-ment†  (2) | Task Difficulty†  (2) | Task Effort†  (2) |
| --- | --- | --- | --- | --- | --- | --- | --- | --- |
| Activity Rate (/min) (1) | .71^**^ | -0.33 | 0.28 | 0.18 | 0.09 | 0.07 | 0.29 | 0.43 |
| N Swaps (1) | -0.38 | .80^***^ | -0.24 | -.52^*^ | 0.09 | -0.08 | -0.45 | -0.34 |
| N Transitions (1) | 0.40 | -0.29 | .60^**^ | 0.21 | -0.13 | -0.05 | 0.00 | 0.28 |
| % Time in  Peaked States (1) | .60^*^ | -.71^**^ | 0.45 | .84^***^ | -0.07 | -0.05 | .64^**^ | .61^*^ |
| Enjoyment† (1) | 0.07 | 0.20 | -0.22 | -0.10 | 0.47 | 0.40 | -0.07 | 0.16 |
| Engagement† (1) | 0.16 | 0.02 | 0.04 | -0.12 | -0.02 | .73^**^ | 0.04 | -0.05 |
| Task Difficulty† (1) | .54^*^ | -.62^**^ | 0.40 | 0.41 | -0.08 | 0.00 | .77^**^ | .53^*^ |
| Task Effort† (1) | -0.05 | -0.36 | 0.12 | -0.09 | 0.22 | .60^*^ | 0.04 | 0.47 |

Note: * = *p* < .05; ** = *p* < .01; *** = *p* < .001. † Indicates that items were taken from post-session questionnaire. (1) indicates data sampled from session 1 and (2) indicates data sampled from session 2.
