# Supplementary material for: Modeling strategic use of human computer interfaces with novel hidden Markov models
Source: Front Psychol. 2015 Jul 3;6:919. doi: 10.3389/fpsyg.2015.00919 (PMC4490801; doi:10.3389/fpsyg.2015.00919)
Supplement: Supplementary file 4 [file Table4.DOCX]

***Supplementary Material***

**Modeling Strategic Use of Human Computer Interfaces with Novel Hidden Markov Models**

**Laura J. Mariano^1^*, Joshua C. Poore^1^, David M. Krum^2^, Jana L. Schwartz^1^, William D. Coskren^1^, Eric M. Jones^1^**

^1^The Charles Stark Draper Laboratory, Inc., Cambridge, MA, USA

^2^University of Southern California, Institute for Creative Technologies, Playa Vista, CA, USA

*** Correspondence:** Laura J. Mariano, The Charles Stark Draper Laboratory, 555 Technology Square, Cambridge, MA, 02139, USA.

[lmariano@draper.com](mailto:lmariano@draper.com)

Table S4

Correlations between task and post-task measures, aggregated across sessions.

| Task-Related  Measures | Activity Rate (/min) | N Swaps | N Transitions | % Time in  Peaked  States | p(Trans. Between Peaked/  Diffuse) | p(Trans. Peaked  to  Peaked) |
| --- | --- | --- | --- | --- | --- | --- |
| Enjoyment† | .05 | .24 | -.31 | -.16 | -.37 | -.27 |
| Engagement† | .13 | .02 | -.06 | -.16 | .11 | -.17 |
| Task Difficulty† | .57^*^ | -.62^**^ | .40 | .62^**^ | .07 | .21 |
| Task Effort† | .21 | -.50^*^ | .27 | .31 | .09 | .25 |

Note: * = *p* < .05; ** = *p* < .01; *** = *p* < .001. † Indicates that items were taken from post-session questionnaire
