# Supplementary material for: Modeling strategic use of human computer interfaces with novel hidden Markov models
Source: Front Psychol. 2015 Jul 3;6:919. doi: 10.3389/fpsyg.2015.00919 (PMC4490801; doi:10.3389/fpsyg.2015.00919)
Supplement: Supplementary file 6 [file Table6.DOCX]

***Supplementary Material***

**Modeling Strategic Use of Human Computer Interfaces with Novel Hidden Markov Models**

**Laura J. Mariano^1^*, Joshua C. Poore^1^, David M. Krum^2^, Jana L. Schwartz^1^, William D. Coskren^1^, Eric M. Jones^1^**

^1^The Charles Stark Draper Laboratory, Inc., Cambridge, MA, USA

^2^University of Southern California, Institute for Creative Technologies, Playa Vista, CA, USA

*** Correspondence:** Laura J. Mariano, The Charles Stark Draper Laboratory, 555 Technology Square, Cambridge, MA, 02139, USA.

[lmariano@draper.com](mailto:lmariano@draper.com)

Table S6

Cross Correlations Between Intake Measures and Session 2 Task-Related Measures

| Intake  Self-Report  Measures | Activity Rate (/min) | N Swaps | N Transitions | % Time in  Peaked  States | Enjoy-  ment† | Engage-ment† | Task Difficulty† | Task Effort† |
| --- | --- | --- | --- | --- | --- | --- | --- | --- |
| Analytic Problems | 0.04 | 0.26 | -0.36 | -0.20 | 0.16 | -0.10 | -0.20 | -0.42 |
| Subjective Numeracy | 0.24 | -0.17 | -0.09 | 0.22 | .53^*^ | -0.06 | 0.32 | 0.43 |
| Cog. Reflections Test | 0.03 | -0.24 | -0.34 | 0.08 | -0.09 | -0.17 | 0.08 | 0.01 |
| Need for Cognition (NFC) | 0.14 | 0.09 | -0.32 | 0.00 | .63^*^ | -0.17 | 0.12 | 0.28 |
| Need For Closure (NFCL) | 0.24 | -0.32 | 0.10 | 0.43 | -.54^*^ | 0.21 | 0.25 | 0.13 |
| Experiential Cog. Style (REI) | -0.05 | -0.21 | -0.03 | 0.01 | 0.27 | 0.33 | -0.24 | .56^*^ |
| Rational Cog. Style (REI) | 0.08 | 0.21 | -0.24 | 0.00 | 0.53 | -0.43 | 0.08 | 0.11 |
| Maximization Scale | -0.09 | 0.11 | -0.15 | -0.37 | 0.14 | -0.10 | -0.11 | 0.36 |

Note: * = *p* < .05; ** = *p* < .01; *** = *p* < .001. † Indicates that items were taken from post-session questionnaire
