# Supplementary material for: Modeling strategic use of human computer interfaces with novel hidden Markov models
Source: Front Psychol. 2015 Jul 3;6:919. doi: 10.3389/fpsyg.2015.00919 (PMC4490801; doi:10.3389/fpsyg.2015.00919)
Supplement: Supplementary file 7 [file Table7.DOCX]

***Supplementary Material***

**Modeling Strategic Use of Human Computer Interfaces with Novel Hidden Markov Models**

**Laura J. Mariano^1^*, Joshua C. Poore^1^, David M. Krum^2^, Jana L. Schwartz^1^, William D. Coskren^1^, Eric M. Jones^1^**

^1^The Charles Stark Draper Laboratory, Inc., Cambridge, MA, USA

^2^University of Southern California, Institute for Creative Technologies, Playa Vista, CA, USA

*** Correspondence:** Laura J. Mariano, The Charles Stark Draper Laboratory, 555 Technology Square, Cambridge, MA, 02139, USA.

[lmariano@draper.com](mailto:lmariano@draper.com)

Table S7

Cross Correlations Between Intake Measures and Task-Related Measures, Averaged Across Sessions

| Intake  Self-Report  Measures | Activity Rate (/min) | N Swaps | N Transitions | % Time in  Peaked  States | Enjoy† | Engage† | Task Diff.† | Task Effort† | p(Trans. Between Peaked/  Diffuse) | p(Trans. Peaked  to  Peaked) |
| --- | --- | --- | --- | --- | --- | --- | --- | --- | --- | --- |
| Analytic Problems | 0.10 | 0.35 | -0.32 | -0.25 | -0.01 | -0.04 | -0.22 | -0.35 | 0.33 | -0.40 |
| Subjective Numeracy | 0.27 | -0.31 | -0.12 | 0.26 | .52^*^ | -0.09 | 0.31 | 0.23 | -.49^*^ | -0.12 |
| Cog. Reflections Test | 0.08 | -0.27 | -0.30 | 0.01 | 0.07 | -0.24 | -0.06 | -0.14 | -0.43 | -0.21 |
| Need for Cognition (NFC) | 0.19 | 0.05 | -0.33 | 0.06 | .72^**^ | -0.11 | -0.15 | 0.04 | -.75^**^ | -0.22 |
| Need For Closure (NFCL) | 0.26 | -0.26 | 0.10 | 0.36 | -0.47 | 0.23 | 0.37 | -0.01 | .55^*^ | -0.08 |
| Experiential Cog. Style (REI) | 0.11 | -0.17 | -0.05 | 0.07 | 0.32 | 0.25 | -0.21 | 0.47 | -0.08 | 0.03 |
| Rational Cog. Style (REI) | 0.11 | 0.16 | -0.23 | 0.06 | .619^*^ | -0.33 | -0.15 | -0.12 | -.77^**^ | -0.11 |
| Maximization Scale | 0.08 | 0.15 | -0.11 | -0.35 | 0.10 | -0.01 | -0.12 | 0.03 | 0.21 | -0.07 |

Note: * = *p* < .05; ** = *p* < .01; *** = *p* < .001. † Indicates that items were taken from post-session questionnaire
